# Financial Benefits of the Early Fitting of a Cochlear Implant Speech Processor: Assessment of the Direct Cost

**DOI:** 10.7759/cureus.5684

**Published:** 2019-09-17

**Authors:** Eman A Hajr, Fida Almuhawas

**Affiliations:** 1 Department of Ear, Nose and Throat, Imam Mohammad Ibn Saud Islamic University, Riyadh, SAU; 2 Otolaryngology, King Abdullah Ear Specialist Center, Riyadh, SAU

**Keywords:** cochlear implant, cost evaluation, direct costs

## Abstract

Objective

Cochlear implants (CIs) are typically activated four weeks after the implantation surgery. This delay between device implantation and activation lengthens the implant process and consequently induces personal and financial burdens for some patients who travel from remote regions to receive the surgery. However, fitting the speech processor and eliminating the waiting period could decrease the indirect cost associated with cochlear implantation. The objective of this study was to assess the impact of an early CI fitting on the overall cost paid by patients and their families aiming to improve future care strategies for patients receiving CIs.

Methods

This retrospective study was conducted in a tertiary referral center. All patients who received any kind of CI with early fitting of the speech processor were included. The total financial benefit for the patients and their families over the standard activation visit was investigated by assessing the cost of the non-medical expense for one hospital visit.

Results

Our results showed that the non-medical cost for each hospital visit associated with cochlear implantation was higher for those who traveled from remote areas: 81 USD for each patient within 200 km of the implantation center and 748.56 USD for each patient farther than 200 km from the implantation center.

Conclusions

Using the early fitting approach, some of the financial burden associated with implantation could be alleviated.

## Introduction

Cochlear implants (CI) are widely regarded as one of the greatest advances of modern medicine, already greater than the combined populations of individuals receiving any other neural prosthetic. The number of patients receiving CIs worldwide continues to increase [1]. In addition, the technology used in CIs has made substantial progress with respect to the improvement of CI outcomes, and various surgical techniques have been developed to enable smaller incisions, minimize complications, promote faster wound healing, and therefore promote patient satisfaction [2-5].

As a common practice, the CI is activated at three to six weeks post-implantation. This waiting period is recommended to encourage proper healing of the surgical site and to allow any swelling to subside [6]. Hence, patients need to visit the hospital once more after CI surgery to be fitted with a speech processor. This could lead to personal and financial burdens for those patients. Alternatively, early fitting of the speech processor (fitting and activating on the day after surgery prior to discharge) could decrease both the waiting period and the number of hospital visits for those individuals; this could further decrease non-medical costs associated with cochlear implantation, such as accommodation and transportation expenses.

Our institution is considered one of the largest centers specialized in ear surgery and related services in the world. Each year, we receive a large number of referred patients from multiple regions across the Kingdom of Saudi Arabia (KSA), as well as from surrounding countries. Hence, patients typically travel to our center from remote regions to receive their implant(s). As the efficacy and safety of the early fitting of the speech processor have been previously demonstrated, this study sought to assess the benefit of this approach on reducing the overall cost of non-medical expenses associated with CI [7].

## Materials and methods

We retrospectively reviewed the charts of all patients who received their CIs from January 2016 to December 2017. All patients who received implants according to the early fitting approach were included regardless of whether they received unilateral or bilateral CI. A total of 100 patients were identified.

The use of human participants in this study was reviewed and approved by the Internal Review Board of the College of Medicine Research Center (E-14-1180). A phone-based questionnaire that included a request to obtain consent to use their data for this research was administered to the families of all participating patients. Specifically, data related to the overall cost associated with implantation were collected in order to analyze the cost-effectiveness of using early fitting; this included information about the patients' residency, the average cost of travel by air or land, per diem cost of accommodation, number of family members who accompanied the patient, duration of the stay needed for each visit, type and cost of transportation to and from the hospital, and the average expenses per day. While most of the families were motivated and willing to participate and to share their data with our research team in order to provide the best care for future CI candidates, 19 eligible patients were excluded from this study: three were unreachable at the phone number recorded in their files, and 16 refused to participate in this study. The final sample thus included 81 patients.

**Figure 1:**
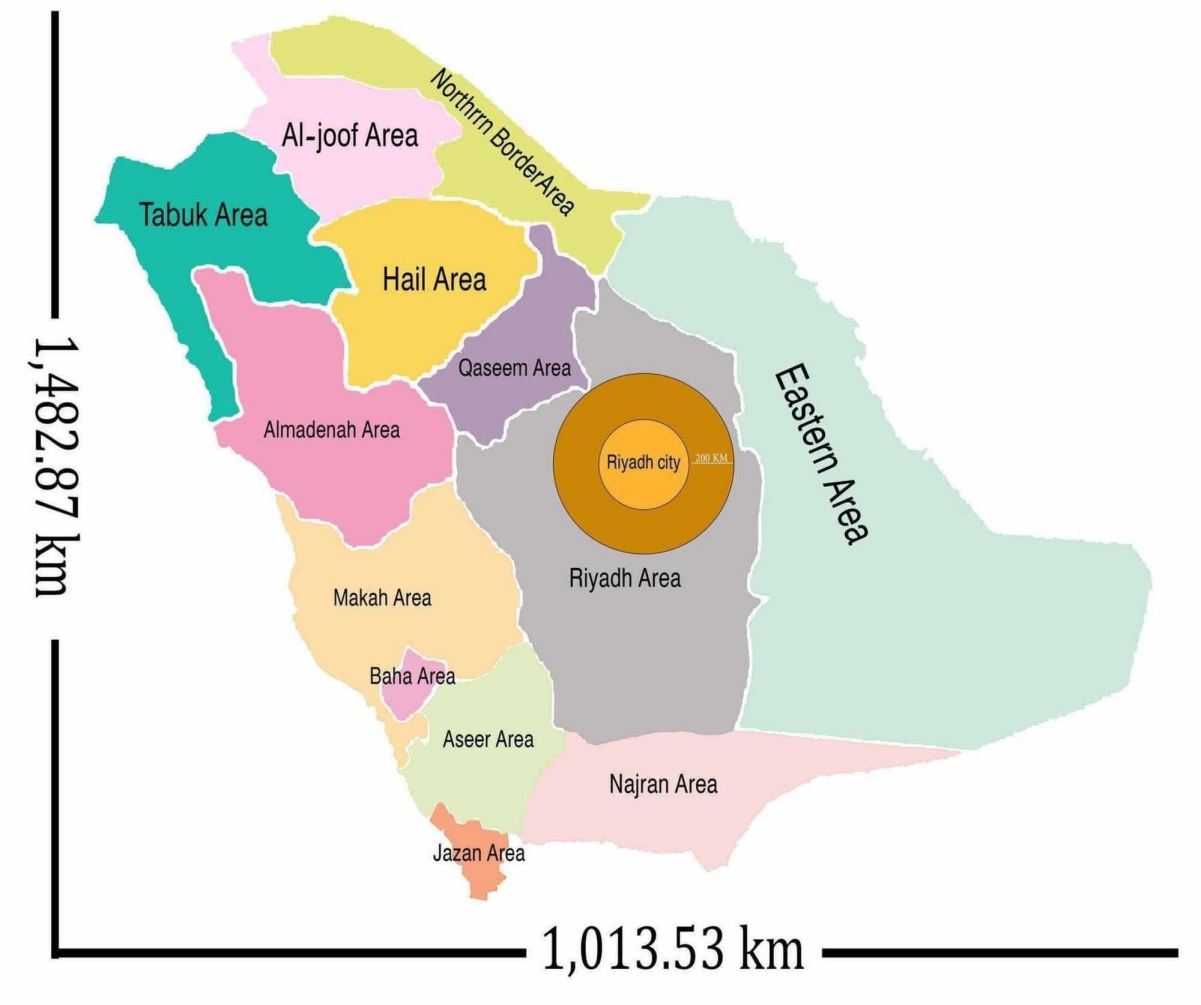
Administrative regions of the Kingdom of Saudi Arabia and the distance for that region from Riyadh (the city of the implant center)

To ensure the homogeneity of the data concerning the cost analysis, patients were divided into three groups based on their place of residency and the distance from the city of the implant center: group 1 (resident in the city where the implant center is situated), group 2 (resident near the city of the implant center, up to a distance of 200 km), and group 3 (resident in a city situated at least 200 km from the implant center, a distance more than 200 km). Figure 1 shows the administrative regions comprising the KSA and illustrates their respective distances from the city where the implant center is located.

Data analysis was conducted using SPSS Statistics, version 24 (SPSS Inc, Chicago, IL). Quantitative variables were presented as the mean with the standard deviation or as the median and range. Qualitative variables were presented as frequencies and percentages. The Kolmogorov-Smirnov test was performed to determine the normality of the quantitative variables. Parametric variables were compared between two groups using an independent sample t-test. Non-parametric variables were compared between two groups using the Mann-Whitney test.

## Results

**Figure 2:**
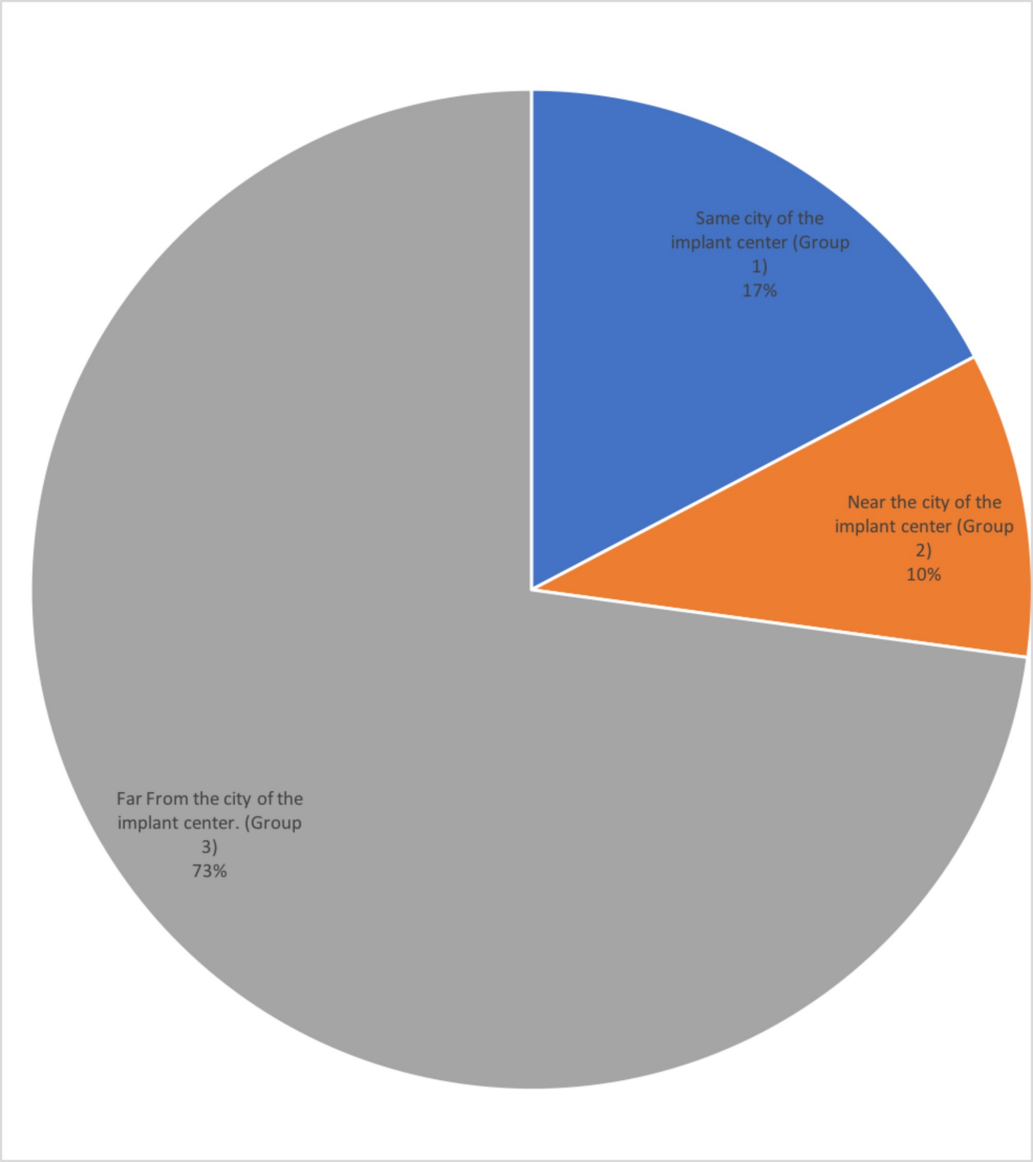
Distribution of cases according to distance from the implant center

A total of 81 participants received early fitting of speech processor were contacted and completed the phone-based questionnaire. The distribution of participants based on their place of residency is presented in Figure 2, which shows that the majority of our patients (73%) were referred from cities far from the implant center.

Group 1 patients were excluded from cost analysis since they live in the same city as the implant center and no additional traveling expense was needed for follow-up visit.

Table 1 shows the mode and the cost of travel to the implant center. All group 2 participants travel by land, while the majority of participants in group 3 (81%) traveled by air. The traveling cost was highest for group 3 participants. Accommodation ranged from 40-120 United States Dollars (USD)/day.

**Table 1:** Traveling costs to the city of the implant center Group 2: Near the implant center; Group 3: Far from implant center; USD: United States dollars; SD: standard deviation

	Patient number	Range (USD)	Mean (USD)	SD (USD)
Group 3 (Air)	48	240-800	475	120
Group 3 (Car)	11	27-333	95	97
Group 2 ( All traveled by car)	8	13-27	20	7

Table 2 shows the accommodation costs, which reflects the preference of patients to stay for 2 to 3 days in the city of the implant center: the day before, of, and after the appointment but in average this will cost around 66 USD /day . This is especially the case if that patient came from a more distant area.

**Table 2:** Distribution of cases according to accommodation cost per day

Cost in United States Dollars	Below average	Average	Above average	Total	Range	Mean	Standard deviation
Number of Cases	25	20	16	61	40-120	66	18
%	41.0	32.8	26.2	100.0			

The overall non-medical related expenses were estimated for all patients. For patients from group 1, the non-medical expenses for follow-up visits were generally negligible because, given the proximity of their residency to the implant center, patients did not have to travel long distances or find accommodation. Correspondingly, the highest expenses were observed for those patients from distant areas. The cost of non-medical expenses for each hospital visit was about 81 USD for each patient in group 2 and 748.56 USD for each patient in group 3 (Table 3); these numbers reflect the average cost for a 1-day accommodation. Therefore, the overall cost is likely to be higher for some individuals depending on the required duration of the stay per visit and the number of accompanying individuals.

**Table 3:** Total difference in cost between the groups

		N	Mean	Standard Deviation	Standard Error of the Mean	p-value	
Total cost in United States Dollars	Group 3: Far from the City of the Implant Center	59	748.56	258.65	33.67	<0.001
	Group 2: Near the City of the Implant Center	8	81.00	33.85	11.97	

## Discussion

CIs represent a category of intervention that is tremendously life-altering. The cost-effectiveness of cochlear implantation in children and adults has been evaluated in the literature [8-11] especially regarding simultaneous or sequential bilateral implants [12-14]. Health insurance coverage is fortunately available in the KSA to cover the direct cost of implantation. However, there are also non-medical related expenses that are typically not included in this coverage, including accommodation and commuting expenses.

In some countries, such as the KSA, there are a limited number of specialized CI centers that can provide comprehensive treatment, which include surgical interventions as well as audiological and rehabilitation management. Hence, patients typically travel to our center from remote regions to receive their implant(s). As a common practice, patients typically wait 3-6 weeks to activate their implants. During this time, patients/caregivers can choose to either stay in the same area of the CI center or to travel back for their activation appointment. This waiting period could increase the non-medical expenses leading to personal and financial burdens for these patients. The overall cost of implantation may thus become a primary factor in the decision to pursue a CI. Searching for new strategies to optimize patient financial satisfaction is crucial to minimizing the likelihood of a patient’s rejection of receiving a CI. 

At our center, we implemented a new approach that aims to activate the speech processor shortly after implantation [15]. Our previous studies have demonstrated that patients can be safely and effectively fitted with their speech processor as early as the day following surgery. Furthermore, early fitting of the speech processor could encourage the acceptance of implantation for these individuals by decreasing both the waiting period and the number of hospital visits, which could diminish the non-medical costs associated with cochlear implantation. Therefore, this study explored the overall cost of these non-medical expenses for different groups of patients who were implanted using the early fitting approach.

As anticipated, this study demonstrated that the cost of non-medical expenses increased with the patient’s distance from the implant center. This is important to take into consideration, especially since 73% of our patients were referred from other regions. As shown in Table 3, patients from the central regions of the KSA spent 81 USD per clinical visit on average; this increased to 748.56 USD per day for patients who were referred from peripheral regions. However, the overall cost can vary significantly depending on the duration of the stay per visit and the number of family members who accompany the patient. By comparison, patients from areas adjacent to the implant center endured very few additional expenses.

Generally, these reported expenses represent the total non-medical expenses associated with CI. However, if the standard fitting approach was used, and patients were required to wait for the activation, this would considerably inflate the indirect cost of implantation because patients/caregivers are required to either stay in the area or travel and come back for their activation appointment after four weeks. This inflation in the indirect cost could exceed the financial capacity of these patients and consequently affect their decision to pursue an implant. By using the early fitting approach, the need for patients returning to the clinic for implant activation is eliminated and, hence, one can expect these expenses to be reduced to the amounts shown in Table 3.

While this report highlighted the indirect cost of CI, the retrospective nature of the study may have compromised the accuracy of our findings; some patients may not have been able to remember the exact cost and instead reported general estimations. Furthermore, this estimation not limited to patients who receive CIs but may also help medical teams and hospital administrations to understand the hardship induced by unnecessary hospital visits, especially concerning patients from remote areas.

## Conclusions

The hardships associated with the CI process could affect patients' decisions to receive an implant, especially for those who must travel from more remote regions. Therefore, early fitting may serve as an effective means of alleviating some of the financial and personal hardships associated with implantation.
